# Vasoactive agents for the prediction of early- and late-onset preeclampsia in a high-risk cohort

**DOI:** 10.1186/1471-2393-13-110

**Published:** 2013-05-12

**Authors:** Pia M Villa, Esa Hämäläinen, Annukka Mäki, Katri Räikkönen, Anu-Katriina Pesonen, Pekka Taipale, Eero Kajantie, Hannele Laivuori

**Affiliations:** 1 The Research Programs Unit, Women’s Health, University of Helsinki, Helsinki, Finland; 2 Department of Obstetrics and Gynaecology, University of Helsinki and Helsinki University Central Hospital, Helsinki, PL140, 00029 HUS, Finland; 3 HUSLAB and Department of Clinical Chemistry, Helsinki University Central Hospital, Helsinki, Finland; 4 Faculty of Behavioural Sciences, Institute of Behavioural Sciences, University of Helsinki, Helsinki, Finland; 5 Iisalmi Hospital, Iisalmi, Finland; 6 Kuopio University Hospital, Kuopio, Finland; 7 Department of Chronic Disease Prevention, National Institute for Health and Welfare, Helsinki, Finland; 8 Hospital for Children and Adolescents, Helsinki University Central Hospital and University of Helsinki, Helsinki, Finland; 9 Haartman Institute, Medical Genetics, University of Helsinki, Helsinki, Finland

**Keywords:** Preeclampsia, Prediction, Vasoactive agents, Placental growth factor, Soluble fms-like tyrosine kinase-1

## Abstract

**Background:**

To evaluate the soluble fms-like tyrosine kinase-1 (sFlt-1), placental growth factor (PlGF), and sFlt-1/PlGF ratio for the prediction of early- and late-onset preeclampsia in a high-risk cohort.

**Methods:**

We studied serial serum samples collected prospectively at 12 + 0 - 14 + 0, 18 + 0 - 20 + 0, and 26 + 0 - 28 + 0 weeks + days of gestation in 6 women who developed early-onset preeclampsia (before 34 weeks of gestation) and in 21 women who developed late-onset preeclampsia (after 34 weeks of gestation) with automated ElecSys 2010 immunoanalyzer (Roche Diagnostics, Germany). Twenty-six high-risk women and 53 women without risk factors with normal pregnancies served as controls.

**Results:**

Serum PlGF concentrations were lower at 18 + 0 to 20 + 0, and 26 + 0 to 28 + 0 weeks of gestation in women who developed early-onset preeclampsia compared to women who developed late-onset preeclampsia and to controls (p < 0.05 for all comparisons). At 18 + 0 to 20 + 0 weeks of gestation area under the receiver-operating characteristic curve (AUC) for serum PlGF was 99.8% (p = 0.0007, 95% CI 99.0-100.0). At 26 + 0 to 28 + 0 weeks of gestation serum sFlt-1/PlGF ratio explicitly detects those women who developed early-onset preeclampsia (AUC 100.0%, p = 0.0007, 95% CI 100–100). Amongst women with late-onset preeclampsia, those who developed severe form of the disease (N = 8) had significantly higher serum sFlt-1 concentrations at all three timepoints (p = 0.004, p = 0.006, and p = 0.003, respectively) compared to women with non-severe form (N = 13).

**Conclusions:**

Low serum PlGF concentration predicts early-onset preeclampsia from the second trimester and elevated serum sFlt-1/PlGF ratio from 26 to 28 weeks of gestation. Elevated serum sFlt-1 concentration in the first trimester in women who later develop late-onset, severe preeclampsia may suggest different etiology compared to the late-onset non-severe form of the disease.

## Background

Preeclampsia is one of the leading causes of maternal and fetal morbidity and mortality. It is a multiorgan disease, defined according to new onset hypertension and proteinuria developing after 20 weeks of gestation [1]. Although the pathogenesis of the disorder starts much earlier in pregnancy, and a number of known clinical risk factors exist (e.g. obesity, primiparity, and a history of preeclampsia in multiparous women), there is no established way to predict the disorder in clinical practice.

The etiology and pathogenesis of preeclampsia is not completely understood, but it originates in the placenta, and maternal endothelium is the target of the disease [2,3]. Dysregulation and imbalance of placental proangiogenic and antiangiogenic vasoactive agents, soluble fms-like tyrosine kinase 1 (sFlt-1), vascular endothelial growth factor (VEGF), and placental growth factor (PlGF) play a significant role in the pathogenesis [4-6].

In the early first trimester of normal pregnancy sFlt-1 concentration is over tenfold and PlGF over twofold higher than in non-pregnant state. In pregnancies ending in early miscarriage both sFlt-1 and PlGF concentrations, measured at gestational weeks six to ten, are significantly lower than in pregnancies with live birth [7]. After the first trimester sFlt-1 stays constant and PlGF increases until the end of the second trimester. During the last two months of a normotensive pregnancy the level of sFlt-1 increases and the level of PlGF decreases in maternal blood [8,9]. These changes occur earlier and are more pronounced in women who will develop preeclampsia [8]. Moreover, these changes may be further exaggerated in pregnancies with an early-onset of preeclampsia, and in preeclamptic pregnancies associated with small for gestational age fetus [8]. Therefore, these vasoactive agents may be promising biomarkers for predicting preeclampsia in early gestation. According to earlier studies PlGF concentrations may be significantly lower already from the late first trimester in women who will developed preeclampsia. Changes in the sFlt-1 concentrations occur later [10]. Accurate prediction of preeclampsia would be essential to identify women who benefit most from intensive monitoring allowing rapid intervention with therapeutic procedures when necessary. Low dose aspirin, started before 16 weeks of gestation, may be effective in prevention, or at least delay the onset of early-onset and severe preeclampsia in high-risk women [11,12]. However, it is not known who would benefit most from aspirin treatment.

We hypothesize that PlGF and sFlt-1 can be used as markers in the prediction of pre-eclampsia. Our aim was to study concentrations of sFlt-1 and PlGF and their ratio (sFlt-1/PlGF) in prospectively collected serial serum samples in a cohort of pregnant women with clinical risk factors for preeclampsia, and controls with special reference to early- and late-onset disease.

## Methods

### The PREDO project

This nested case–control study is a part of a multidisciplinary PREDO Project (Prediction and Prevention of Preeclampsia) approved by the Ethics Committee of Obstetrics and Gynecology Hospital District of Helsinki and Uusimaa. The study cohort was collected prospectively between September 2005 and June 2009 in ten participating hospital maternity clinics. The PREDO Project has been previously described in detail [11]. Briefly, we recruited 947 pregnant women with clinical risk factors for preeclampsia, and 117 pregnant women without known clinical risk factors for preeclampsia served as a control group. The recruitment took place at the time of the first ultrasound screening at 12 + 0 to 14 + 0 (weeks + days) of gestation in one of ten Finnish hospital maternity clinics participating in the PREDO Project; Women’s Hospital, Midwifery Institute (Kätilöopisto) Maternity hospital and Jorvi Hospital at Helsinki University Central Hospital, Kanta-Häme Central Hospital, Päijät-Häme Central Hospital, Tampere University Hospital, Kuopio University Hospital, Northern Karelia Central Hospital, Hyvinkää Hospital and Iisalmi Hospital. All women with a bilateral diastolic notch in the uterine artery Doppler velocimetry at 12–14 weeks of gestation were included in the medication group (n = 152) and randomised to acetylsalicylic acid or placebo. All of these women gave blood samples for biochemical analyses at three timepoints during the pregnancy. The other women for venipuncture were selected randomly: after a woman was included in medication group the next participant recruited with risk factors (without aforementioned ultrasound finding) gave blood samples at three timepoints during the pregnancy. A written informed consent was obtained from all participants before entering the study.

### Data collection and diagnosis of preeclampsia

Pregnancy data were collected from the medical records of maternity clinics and hospitals. Pregnancy outcomes were ascertained before biochemical analyses by a jury of two medical doctors and a midwife who met face-to-face and agreed upon the diagnosis of each participant based on criteria described above.

### Subcohort for serum sFlt-1 and PlGF measurements

We included in this substudy 26 women with clinical risk factors for preeclampsia who eventually developed the disease. All women whose preeclampsia diagnosis was ascertained by the jury at the time these biochemical analyses were started, were included. Twenty-six women with clinical risk factors but without preeclamptic pregnancy, chosen by computerized randomization, formed a first control group. Fifty-three women without clinical risk factors and without preeclamptic pregnancy formed a second control group. One woman without clinical risk factors developed preeclampsia and she was included in the group of women with preeclampsia. Preeclamptic women were categorized by the onset and severity of the disease.

All included women were Caucasian. Definitions and the inclusion criteria for the PREDO Project are presented in Table 1. Severe preeclampsia was defined as systolic blood pressure ≥ 160 mmHg and/or diastolic ≥ 110 mmHg in two consecutive measurements and/or proteinuria > 5 g in 24 hour urine collection.

**Table 1:** Inclusion criteria and definitions

**Inclusion criteria**	**Early-onset preeclampsia n = 6**	**Late-onset preeclampsia n = 21**	**Women with risk factors n = 26**
Complications in previous pregnancies:			
Preeclampsia	1 (17%)	11 (55%)	10 (38%)
(Blood pressure ≥ 140 systolic and/or ≥ 90 mmHg diastolic in two consecutive measurements and proteinuria ≥0.3 g/in 24-hour urine collection or two random urine containing ≥ 1+ by dipstick or one dipstick demonstrating ≥ 2+ protein)			
Fetal growth restriction	3 (50%)	0 (0%)	5 (19%)
(Birthweight SD score < −2 standard deviations (SD) according to the Finnish standards*)			
Fetal death	-	1 (5%)	-
(>22 weeks of gestation or over 500 g)			
A history of one of the following conditions:			
Obesity	2 (33%)	11 (55%)	11 (42%)
(BMI over 30 kg/m^3^ prior to pregnancy)			
Gestational diabetes	-	3 (15%)	3 (12%)
(one or more abnormal values in the oral glucose tolerance test)			
Chronic hypertension	-	4 (20%)	3 (12%)
(≥140/90 mmHg or medication for hypertension before 20th weeks of gestation)			
Age over 40 years	-	1 (5%)	1 (4%)
Age under 20 years	-	-	1 (4%)

Inclusion criteria in the women with risk factors in the ’Prediction and Prevention of PreEclampsia’ (PREDO) Project.

1. Early preeclampsia is diagnosed before 34 + 0 weeks of gestation.

2. Late onset preeclampsia is diagnosed after 34 + 0 weeks of pregnancy.

3. Additional inclusion criteria were Sjögren´s syndrome, systemic lupus erythematosus, type I diabetes which however were present in none of the participants.

4. Exclusion criteria were asthma, allergy to aspirin, tobacco smoking during pregnancy, previous peptic ulcer, placental ablation in a previous pregnancy, inflammatory bowel disease (Crohn’s disease, ulcerative colitis), rheumatoid arthritis, bleeding disorder, trombophilia (previous venous or pulmonary thrombosis and/or coagulation abnormality), or multiple pregnancy.

* According to the Finnish standards by Pihkala et al. Duodecim 1989 (13).

We were able to rule out smoking as a confounding factor, since it was an exclusion criteria for the high risk women. For those women in the second control group, who did not have known risk factors for preeclampsia, smoking was not an exclusion criteria. However, all women filled in a questionnaire concerning their medical history. According to that, from the fifty-three women in the second control group, six smoked during the index pregnancy.

### Sample collection and assays

Blood samples were drawn from antecubital vein at 12 + 0 to 14 + 0, 18 + 0 to 20 + 0, and 26 + 0 to 28 + 0 weeks of gestation. After centrifugation serum samples were stored at - 80°C until the analysis. Serum sFlt-1 and PlGF were performed with automated ElecSys 2010 immunoanalyzer utilizing new ElecSys sFlt-1 and PlGF assays (Roche Diagnostics, Germany). In our laboratory the intra-assay coefficient of variation (CV) for serum sFlt-1 was 2.8%, calculated from duplicate patient samples in the concentration range of 300–11 000 pg/ml (n = 30), and the inter-assay CV < 1.4% (assay control samples, range 880 to 9100pg/ml). For serum PlGF the intra-assay CV was 2,3% (n = 30, concentration range 16–500 pg/ml) and the interassay CV < 1.9% (83-1960 pg/ml).

### Statistical analysis

We analysed data with the SPSS 19.0 program. For comparisons between groups Students t-test and ANOVA were used as appropriate. Bonferroni adjustment was used for multiple comparisons. Since the sizes of the groups differed, we used nonparametric Kruskal-Wallis test, and for corrected: post hoc test pairwise comparisons with adjusted significance, to compare concentrations (PlGF and sFlt-1) and sFlt-1/PlGF ratio between the four groups.

For comparison of sequential changes of PlGF and sFlt-1 concentrations, and sFlt-1/PlGF ratio between groups from first to second, and from second to third measurement, the change of the concentration or ratio between measurements was calculated for each individual. For comparison of these changes between the four groups we used nonparametric Kruskal-Wallis test. Receiver-operating characteristic (ROC) analysis was performed to determine the predictive value of PlGF and sFlt-1/PlGF ratio at 18 + 0 to 20 + 0, and 26 + 0 to 28 + 0 weeks of gestation.

## Results

Six women developed early-onset preeclampsia (onset before 34 weeks of gestation), and twenty-one women developed late-onset disease (onset after 34 weeks of gestation). In the late-onset group, eight women were diagnosed with severe, and thirteen with non-severe preeclampsia. In the early-onset group all six women developed severe form of the disease.

The clinical characteristics of the study groups are presented in Table 2.

**Table 2:** Baseline and pregnancy characteristics

	**Early-onset preeclampsia n = 6**	**Late-onset preeclampsia n = 21**	**Control women with risk factors for preeclampsia n = 26**	**Healthy control women without risk factors for preeclampsia n = 53**
Age at entry, years (SD)	31.2 (3.7)	32.6 (5.0)	29.7 (6.0)	30.1 (4.6)
Nulliparous, n (%)	3 (50%)	3 (17%)*	9 (35%)	34 (64%)
Body mass index before pregnancy, kg/m^3^ (geometric mean and 95% CI)	28.5 (21.8-37.2)**	30.3 (27.1-33.7)**	27.8 (25.5-30.3)**	22.7 (22.0-23.4)
Height, cm (SD)	162.5 (3.3)	166.1 (4.8)	166.3 (6.6)	167.0 (5.5)
Systolic blood pressure, mmHg (SD)	183 (15)**	167 (16)**	140 (18)**	125 (13)
Diastolic blood pressure, mmHg (SD)	111 (11)**	108 (8)**	91 (11)**	81 (7)
Proteinuria g/24 hours urine collection (SD)	5.7 (4.8)	1.6 (2.0)	-	-
Gestational age at delivery, weeks (geometric mean and 95% CI)	32.8 (31.0-34.7)	39.2 (38.6-39.7)^‡^	40.0 (39.6-40.3)^‡^	40.2 (39.8-40.6)^‡^
Birth weight, g (SD)	1600 (490)	3360 (110)^‡^	3480 (470)^‡^	3460 (410)^‡^
Birth weight sd score	−2.2 (0.7)	−0.3 (1.1)^‡^	−0.2 (1.1)^‡^	−0.3 (0.8)^‡^
Gestational age at first sample, weeks (SD)	12.6 (0.6)	12.9 (0.6)	12.8 (0.7)	12.9 (0.6)
Gestational age at second sample, weeks (SD)	19.3 (0.3)	18.9 (0.5)	19.1 (0.6)	19.1 (0.6)
Gestational age at third sample, weeks (SD)	26.3 (0.5)	26.3 (0.6)	26.8 (0.8)	26.6 (0.8)

Continuous, normally distributed data presented as mean (standard deviation, SD).

Continuous non-normally distributed data presented as geometric mean (95% confidence interval, CI).

^*^ p < 0.05 between groups.

** p < 0.05 for comparison with control women without risk factors.

^‡^ p < 0.05 for comparison with early onset preeclampsia group.

Early-onset preeclampsia is diagnosed before 34 + 0 weeks and days of gestation.

Late-onset preeclampsia is diagnosed after 34 + 0 weeks and days of gestation.

There were no significant differences in the gestational age at sampling between the four groups. In the early-onset preeclampsia group the third samples were drawn at 26.0-26.9 weeks of gestation, and diagnoses of preeclampsia were made at 30.7-33.3 weeks of gestation. We excluded the measurement at 27 weeks of gestation in one woman who was diagnosed with early-onset preeclampsia on the same day the blood sample was drawn. In the other women proteinuria was not evident at the time of blood sampling.

### sFlt-1

**Figure 1:**
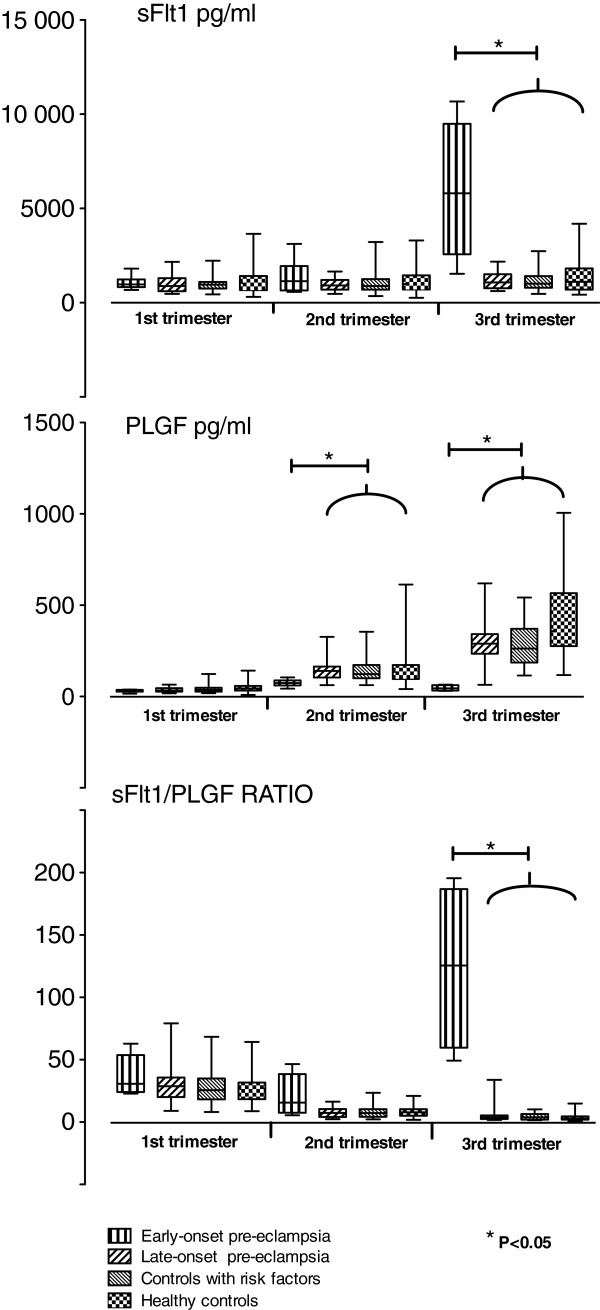
**Box- and whisker plots.** Box- and-whisker plot (mean, 95% CI, range) of sFlt-1 and PlGF concentrations (pg/ml) and sFlt-1/PlGF ratio at 12 + 0-14 + 0, 18 + 0-20 + 0, 26 + 0-28 + 0 weeks of gestation in early-onset and late-onset preeclampsia groups and in controls with risk factors and in healthy controls in the ’Prediction and Prevention of Preeclampsia’ (PREDO) project.

Serum sFlt-1 concentration was significantly higher in women who later developed early-onset preeclampsia compared to women who developed late-onset preeclampsia and to women in the two control groups at 26 + 0 to 28 + 0 weeks of gestation (Table 3). There were no differences between women with late-onset preeclampsia and control groups at any time point (Figure 1). Those women who developed late-onset, severe preeclampsia (n = 8) had significantly higher sFlt-1 concentration than women who were diagnosed with late-onset, non-severe (n = 13) form of the disease at 12 + 0 to 14 + 0, 18 + 0 to 20 + 0, and 26 + 0 to 28 + 0 weeks of gestation (Table 4). In the late-onset preeclampsia subgroup birthweight standard deviation (SD), calculated according to Finnish standards [13], was significantly lower in women with severe form of the disease compared to women with non-severe form (birth weight SD −1.0 (SD 0.9), 0.2 (SD 1.0) p = 0.01 respectively). The body mass index (BMI) was significantly higher in women who developed late-onset non-severe, preeclampsia, compared to women who developed late-onset, severe preeclampsia (BMI 33.6 (SD 7.0), 26.9 (SD 5.8), p = 0.03, respectively).

**Table 3:** Concentrations of placental growth factor, soluble vascular endothelial growth factor receptor-1 and their ratio in three different timepoints during pregnancy

**sFlt-1 pg/ml**				
Weeks + days of gestation	Early-onset preeclampsia	Late-onset preeclampsia	Controls with risk factors	Healthy controls
12 + 0 to 14 + 0	1006.7 (715.0-1417.8)	924.5 (760.3-1124.4)	930.7 (803.2-1078.4)	1020.0 (875.0-1189.0)
18 + 0 to 20 + 0	1147.1 (592.2-2221.3)	918.1 (772.9-1090.7)	922.1 (743.4-1143.9)	969.8 (831.8-1128.3)
26 + 0 to 28 + 0	4847.3* (1318.6-17819.7)	1054.4 (887.2-1252.9)	1034.9 (876.2-1222.4)	1141.3 (975.9-1334.8)
**PlGF pg/ml**				
Weeks + days of gestation	Early-onset preeclampsia	Late-onset preeclampsia	Controls with risk factors	Healthy controls
12 + 0 to 13 + 6	29.4 (21.0-41.1)	35.3 (30.3-41.3)	37.9 (31.6-45.5)	40.9 (35.3-47.3)
18 + 0 to 20 + 0	71.9* (52.8-98.1)	136.7 (112.7-165.8)	132.4 (113.8-154.0)	137.3 (120.9-155.9)
26 + 0 to 28 + 0	44.6* (25.6-77.8)	274.2 (222.4-338.1)	271.5 ‡ (224.9-327.6)	383.8 (332.3-443.3)
**sFlt-1/PlGF ratio**				
Weeks + days of gestation	Early-onset preeclampsia	Late-onset preeclampsia	Controls with risk factors	Healthy controls
12 + 0 to 13 + 6	34.4 (22.2-53.3)	26.6 (21.3-33.2)	24.8 (19.7-30.5)	25.0 (22.3-28.0)
18 + 0 to 20 + 0	15.9 (6.6-38.8)	6.7 (5.2-8.7)	6.9 (5.3-8.9)	7.1 (6.1-8.2)
26 + 0 to 28 + 0	108.8* (40.5-292.1)	4.0 (3.0-5.4)	3.8 (3.0-4.9)	3.0 (2.5-3.6)

Concentrations (geometric mean, 95% confidence interval) of placental growth factor (PlGF) and soluble vascular endothelial growth factor receptor-1 (sFlt1), and sFlt1/PlGF ratio at 12 + 0 to 14 + 0, 18 + 0 to 20 + 0, and 26 + 0 to 28 + 0 weeks of gestation in early-onset (N = 6), late-onset preeclampsia (N = 21), in controls with risk-factors (N = 26), and in healthy controls without known risk-factors (N = 53).

* p < 0.05 compared to late onset preeclampsia, controls with risk factors and heathy control groups.

‡ p < 0.05 compared to healthy controls.

Early-onset preeclampsia is diagnosed before 34 completed weeks of gestation.

Late-onset preeclampsia is diagnosed after 34 completed weeks of gestation.

**Table 4:** Serum sFlt-1 concentration (pg/ml) (geometric mean, 95% confidence interval) at 12 + 0 - 14 + 0, 18 + 0 - 20 + 0, and 26 + 0 - 28 + 0 weeks of gestation in women with late-onset preeclampsia group (onset ≥ 34 + 0 weeks of gestation)

**Weeks + days of gestation**	**Late-onset pre-eclampsia n = 21**	**Severe late-onset preeclampsia n = 8**	**Non-severe late-onset preeclampsia n = 13**
12 + 0 – 14 + 0	925 (760–1124)	1330* (990–1670)	810 (620–1000)
18 + 0 – 20 + 0	918 (773–1091)	1220* (950–1490)	810 (650–980)
26 + 0 – 28 + 0	1054 (887–1253)	1480* (1080–1870)	920 (750–1090)

Severe preeclampsia was defined as systolic blood pressure ≥ 160 mmHg and/or diastolic ≥ 110 mmHg in two consecutive measurements and/or proteinuria > 5 g in 24 hour urine collection.

* p < 0.05 compared to late-onset non-severe preeclampsia subgroup.

The sequential changes in the sFlt-1 concentrations between groups were not significant between the first and the second measurements. The sequential change of sFlt-1 concentration was significantly higher in the early-onset preeclampsia group compared to the other groups between the second and third measurements (p = 0.002).

### PlGF

Serum PlGF concentration was significantly lower in women who went on to develop early-onset preeclampsia compared to the other groups at 18 + 0 to 20 + 0, and at 26 + 0 to 28 + 0 weeks of gestation (Table 3). We did not find any differences in the serum PlGF concentration between the study groups at 12 + 0 to 14 + 0 weeks of gestation, or between women with late-onset preeclampsia and the two control groups at any time point (Figure 1). At 26 + 0 to 28 + 0 weeks of gestation the PlGF concentration in controls with risk factors was significantly lower than in controls without risk factors.

There was a significant difference in the sequential changes of the serum PlGF concentration between the first and the second (p = 0.02), and the second and the third (p < 0.001) measurement when the early- onset preeclampsia group was compared with the late-onset and the two control groups. Between the second and third measurement the PlGF concentration increased over 100% in the late-onset preeclampsia group and the two control groups, whereas in the early-onset preeclampsia group it decreased 40%.

### sFlt-1/PLGF ratio

The serum sFlt-1/PlGF ratio at 26 + 0 to 28 + 0 weeks of gestation was significantly higher in the early-onset preeclampsia group compared to the other groups (Table 3). We did not find any differences in the serum sFlt-1/PlGF ratio between the four groups at 12 + 0-14 + 0 or at 18 + 0 to 20 + 0 weeks of gestation, or between the late-onset preeclampsia group and control groups at any time point (Figure 1).

Between first and second measurements the sequential changes between groups were not different. The sequential change of sFlt-1/PlGF ratio in the early-onset preeclampsia group was significantly higher compared to the late-onset preeclampsia and control groups between second and third measurements (p = 0.002).

After adjusting for BMI before pregnancy the differences in sFlt-1, PlGF, or the sFlt-1/PlGF ratio between groups remained unchanged.

We found a negative correlation between birth weight SD score and the serum sFlt-1/PlGF ratio at all timepoints in women who developed preeclampsia. Correlation was strongest at 26 + 0 to 28 + 0 weeks of gestation (Spearman’s correlation coefficient −0.403, p = 0.04; -0.452 p = 0.02, and −0.673, p = 0.0002 at 12 + 0 to14 + 0, 18 + 0 to 20 + 0, and 26 + 0 to 28 + 0 weeks of gestation, respectively). We did not find any correlation between birth weight SD score and the serum sFlt-1/PlGF ratio in the two control groups.

### Receiver operating characteristic analyses

**Figure 2:**
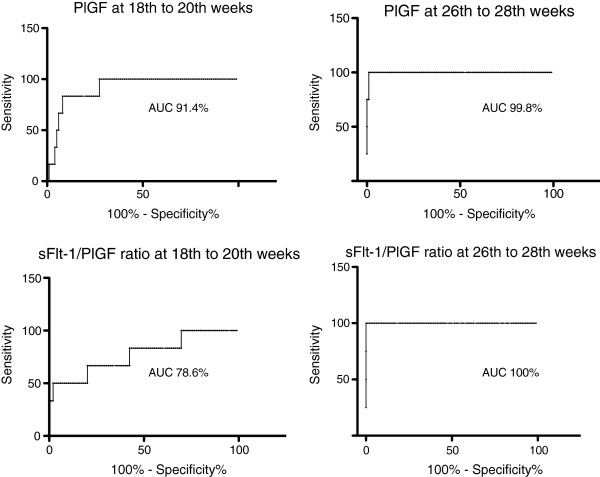
**Receiver operating characteristic curves.** Receiver operating characteristic (ROC) curves in the prediction of early-onset preeclampsia by PlGF and sFlt-1/PlGF ratio at 18 + 0-20 + 0 and 26 + 0-28 + 0 weeks of gestation in the ’Prediction and Prevention of Preeclampsia’ (PREDO) project.

Receiver operating characteristic (ROC) curves (Figure 2) demonstrate the performance of these markers in prediction of early-onset preeclampsia. PlGF (area under the curve (AUC) 91.4%, p = 0.0007, 95% CI 83.4-99.5) has a better predictive value at 18 + 0 to 20 + 0, weeks of gestation than sFlt-1/PlGF ratio (AUC 78.6%, p = 0.02, 56.3-98.9). A cut-off point of PlGF concentration 83.9 pg/ml finds women with future early-onset preeclampsia with a 83.3% sensitivity and 91.1% specificity, with positive predictive value (PPV) 38% and negative predictive value (NPV) 94%. At the 26 + 0 to 28 + 0 weeks of gestation sFlt-1/PlGF ratio performed slighty better than PlGF (AUC 100.0%, p = 0.0007, 100.0-100.0, and 99.8%, p = 0.0008, 99.0-100.5, respectively), and better than sFlt1-1 (AUC 94.4%, p = 0.003, 84.6-100.0). At 26 + 0 to 28 + 0 weeks of gestation all women who developed early-onset preeclampsia and one woman who developed severe late-onset preeclampsia had serum sFlt-1/PlGF ratio over 30. All women who did not develop preeclampsia or who developed late-onset, non-severe form of the disease had sFlt-1/PlGF ratio under 15 at 26 + 0 to 28 + 0 weeks of gestation. If the cut-off point were set to 40, women who later develop early-onset preeclampsia would have been found with 100% sensitivity and specifity (PPV 100%, NPV100%). For PlGF with cut-off point set to 90.4, PPV was 80% and NPV 100%.

## Discussion

In this nested case–control study performed in high risk women we found lower PlGF concentration from 18 + 0 to 20 + 0 weeks of gestation in sera of women who later developed early-onset preeclampsia. Serum sFlt-1/PlGF ratio over 40 at 26 + 0 to 28 + 0 weeks of gestation has high specificity and sensitivity in identifying women who developed early-onset disease. Women who were later diagnosed with late-onset, severe form of preeclampsia had significantly increased levels of sFlt-1 already at 12 + 0 to 14 + 0 weeks of gestation compared to women with late-onset, non-severe form of the disease. This is a new finding in a study with a small sample size and needs to be confirmed in a larger study population.

We would like to emphasize that these findings are likely to be specific to high risk women.

Earlier studies have reported that the ratio of sFlt-1/PlGF is a better predictor of early preeclampsia than the two markers alone [14]. We found that serum sFlt-1/PlGF ratio was significantly higher from 26 + 0 to 28 + 0 weeks of gestation in women who later developed early-onset preeclampsia compared to the three other study groups. However, in our study PlGF alone was a better predictor of early pre-eclampsia at 18 + 0 to 20 + 0 weeks of gestation. In the study of Levine and coworkers [6] sFlt-1/PlGF ratio was significantly higher already from 17 to 20 weeks of gestation in women who developed preterm preeclampsia. Moore Simas and coworkers [15] found that sFlt-1/PlGF ratio measured at 22 to 26 weeks of gestation was highly predictive of early-onset preeclampsia. In these two studies significantly increased sFlt-1/PlGF ratio was also found at 25 to 30 weeks of gestation in women destined to develop late-onset preeclampsia. In contrast, we did not observe significant difference in sFlt-1/PlGF ratio between the late onset preeclampsia group and the two control groups at any of the measured time points. In our study all women who were destined to develop early-onset preeclampsia could be identified at 26 + 0 to 28 + 0 weeks of gestation, 4.0 to 6.3 weeks before the diagnosis of preeclampsia, by sFlt1/PlGF ratio, cut-off point set so, that no false positives existed. In other words, we were able to identify the developing early-onset disease among the high-risk population at least one month before the clinical diagnosis was made. From the clinical perspective, these findings are significant by helping obstetricians to make decisions on the management of the high-risk women; how intensive follow-up is required, whether hospitalization is essential, and whether it is necessary to be prepared to early delivery in a tertiary care center.

Interestingly, recent studies have proposed that sFlt-1/PlGF ratio may be useful not only as a predictor of preeclampsia but also in the differential diagnosis of hypertensive diseases of pregnancy, and, as well, serve as a prognostic parameter in patients with established preeclampsia [16,17]. Verlohren and coworkers proposed that sFlt-1/PlGF ratio may be used in individualized risk stratification in patients with clinical preeclampsia, and clinical management can be adapted accordingly [17]. Rana and coworkers [18], studied angiogenic factors in 616 women with a suspected preeclampsia, and found that in early-onset (<34 weeks) disease sFlt-1/PlGF ratio predicts adverse outcome occurring within two weeks. Even in association with the atypical presentation of preeclampsia, with relatively normal blood pressure or with no proteinuria, sFlt-1/PlGF ratio performed well. Rana and coworkers showed an inverse correlation between sFlt-1/PlGF ratio and the remaining duration of pregnancy.

Many studies have demonstrated low levels of PlGF already in the first trimester in women who later developed preterm or term preeclampsia [10,19-22]. Cowans and coworkers [23] and Noori and coworkers [24] found significantly lower PlGF levels from the first trimester of pregnancy in women who later developed early-onset or preterm preeclampsia, but not in women with term preeclampsia. We found significant differences in the serum PlGF concentrations between the early-onset preeclampsia group and the three other study groups, however, not earlier than 18 + 0 to 20 + 0 weeks of gestation. We showed that at 18 + 0 to 20 + 0 weeks of gestation, 7.3 to 13.4 weeks before the diagnosis, PlGF could predict early-onset preeclampsia in our high-risk population with an AUC 91.4%. The performance of PlGF as a predictive marker improves over the course of pregnancy so that, 26 + 0 to 28 + 0 weeks of gestation, PlGF has AUC 99.8% (PPV 80%, NPV 100%) in predicting early-onset preeclampsia in this study population. However, during the second half of pregnancy, sFlt-1/PlGF ratio performed even better (PPV 100%, NPV 100%).

Our study is in line with most earlier studies in finding significant differences of sFlt-1 concentration between women who are destined to develop preeclampsia and controls not earlier than during the second half of pregnancy [15,20,25]. However, some studies have reported elevated sFlt-1 concentration already from thirteen to twenty weeks of gestation [18,26-28]. Analyses performed using first trimester serum samples have resulted in negative findings [19,21,22]. Vatten and coworkers [29] studied a cohort consisting of 154 women who later developed preterm preeclampsia (diagnosis before 37 weeks of gestation), 190 women with term preeclampsia (diagnosis after 37 weeks of gestation) and 392 control women. They found that sequential change of sFlt-1 concentration between first and second trimester strongly predicts preeclampsia. Moreover, in the preterm preeclampsia group the sequential change in sFlt-1 concentration was steeper than in the term preeclampsia group. One major difference between our study and the studies of others was that we did not find any differences between the late onset preeclampsia and the two control groups at any of the measured time points.

Vasoactive agents may behave differently not only in early-onset and late-onset preeclampsia, but also in severe and non-severe cases. This might reflect the differences in pathogenesis of subtypes of preeclampsia. Early-onset preeclampsia is considered more as a placental disease whereas late-onset more as a maternal disease. Early-onset preeclampsia is considered a consequence of abnormal placentation and it has often a familial predisposition suggesting a genetic component and a high recurrence risk [30]. In the early-onset form of the disease placental insufficiency often results in fetal growth restriction. Late-onset disease emerges from maternal predisposing risk factors, eg. metabolic factors, associated with obesity, chronic hypertension, diabetes, and interacting with a normal placenta [30]. Our finding of significantly higher serum sFlt-1 concentration in maternal serum already from 12 + 0 to 14 + 0 weeks of gestation in women who developed late-onset, severe, preeclampsia compared to women who developed late-onset, non-severe form of the disease could reflect this phenomenon. Women with late-onset, severe preeclampsia gave birth to significantly lighter newborns, whereas women with late-onset, non-severe form of the disease had significantly higher BMI in early pregnancy arguing for a metabolic etiology.

The strength of our study is a well-characterized prospective cohort. However, we acknowledge that the number of women who developed early-onset preeclampsia was small even if we studied women with clinical risk factors for preeclampsia. One limitation of our study is that we do not have serum samples available after 30 weeks of gestation. Therefore we are not able to study how these biomarkers behave close to the diagnosis of late-onset preeclampsia. Our prospective study reflects the true composition of early-onset and late-onset preeclampsia in women at high-risk, which may explain differences between the present and some earlier studies. If the proportion of women with early-onset preeclampsia compared to women with late-onset preeclampsia is higher than usually observed in the clinical practise, and the early- and late-onset preeclampsia groups are analysed together, the results may be biased. Moreover, many preanalytical and methodological differences may exist between studies. These include the different sample material (plasma or serum) and the storage and handling of the samples. The assays may have a different specificity and sensitivity and the study population may differ between studies.

## Conclusions

In conclusion, using serum PlGF we were able to identify already before 20 weeks of gestation women who will develop early-onset preeclampsia. Even more accurately sFlt-1/PlGF ratio identified those women who developed early-onset preeclampsia, weeks before the onset of clinical disease.

The significantly increased serum sFlt-1 concentration already from the 12 weeks of gestation in the late-onset, severe preeclampsia compared to late-onset non-severe preeclampsia suggests for different pathological background in the late-onset subgroup.

## Competing interests

Roche Diagnostics Ltd and Perkin Elmer Ltd paid Dr Pia M Villa’s travel expences to medical meetings held in Germany and in Denmark during year 2012.

## Authors’ contributions

The authors made the following substantial contributions to this work: concept and design: PV, EH, KR, AKP, PT, EK, HL; analysis and interpretation of data: PV, EH, AM, HL; drafting the article: PV, EH, HL; and revising the manuscript for intellectual content: all authors. Final approval of the version to be published was given by all authors.

## Pre-publication history

The pre-publication history for this paper can be accessed here:

http://www.biomedcentral.com/1471-2393/13/110/prepub
